# Allergen alters IL‐2/αIL‐2‐based Treg expansion but not tolerance induction in an allergen‐specific mouse model

**DOI:** 10.1111/all.14203

**Published:** 2020-02-15

**Authors:** Cordula Köhler, Ursula Smole, Bernhard Kratzer, Doris Trapin, Klaus G. Schmetterer, Winfried F. Pickl

**Affiliations:** ^1^ Institute of Immunology Center for Pathophysiology, Infectiology and Immunology Medical University of Vienna Vienna Austria; ^2^ Clinical Department of Medical and Chemical Laboratory Diagnostics Medical University of Vienna Vienna Austria

**Keywords:** allergy treatment, IL‐2/αIL‐2 complexes, immunotherapy and tolerance induction, T cells, Treg cells

## Abstract

**Background:**

Regulatory T lymphocytes (Treg) play an important role in preventing allergic diseases. We characterized Treg expansion kinetics, marker profiles, and recirculation behavior in allergen‐challenged mice, which had been pretreated with IL‐2/αIL‐2 complexes in the presence or absence of allergen. Moreover, the ability of induced Treg to control airway hyperreactivity and effector functions of lung T cells was determined.

**Methods:**

Humanized TCR/HLA‐transgenic allergy mice were treated in vivo with recombinant IL‐2 complexed to the anti‐IL‐2 mAb JES6‐1 in the presence or absence of mugwort pollen extract (MPE) on days 0‐2. Afterward, they were intranasally challenged with MPE (days 13‐15) followed by determination of airway hyperreactivity and lung T cell effector functions. Multiparametric flow cytometry on peripheral blood T cells was performed on a daily basis.

**Results:**

IL‐2/αIL‐2 complexes highly efficiently expanded peripheral Treg cells, while concomitant allergen exposure altered the phenotype of expanded Treg cells. Notably, application of allergen together with IL‐2/αIL‐2 complexes induced expression of Treg marker molecules CTLA4, NRP1, Helios, and GITR on conventional T cells. Apart from CD25, GARP was identified as the most reliable surface‐expressed lineage discrimination marker of Treg expanded in the presence of IL‐2/αIL‐2 complexes and allergen. Finally, IL‐2/αIL‐2 complex‐expanded Treg cells could be recalled upon allergen challenge, which was associated with suppression of lung‐specific Th2 responses long after initial treatment.

**Conclusion:**

The characterization of reliable surface and transcription markers of IL‐2/αIL‐2 complex‐expanded Treg along with their expansion kinetics and function will help to identify protocols for their long‐term expansion in vivo.

## Graphical abstract

Application of IL‐2/αIL‐2 complexes changes the phenotype and function of peripheral blood T cells by inducing a wave of recirculating Treg cells. Presence of MPE changes the expansion kinetics and the phenotype of the expanded Treg population and reveals lack of specificity of several Treg marker molecules. IL‐2/αIL‐2 complex‐induced Tregs can be recalled when mice are allergen challenged on days 13‐15 and protect from allergic lung inflammation long after initial treatment.

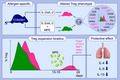

**Abbreviations**
AHR: airway hyperreactivity
APC: antigen‐presenting cell
CD: cluster of differentiation
CTL: cytotoxic T lymphocytes
DC: dendritic cells
FOXP3: forkhead box protein 3
HLA: human leukocyte antigen
IFN: interferon
Ig: immunoglobulin
IL: interleukin
MFI: mean fluorescence intensity
MHC: major histocompatibility complex
MPE: mugwort pollen extract
NK: natural killer cell
PB: peripheral blood
PHA: phytohemagglutinin
RAG: recombination activation gene
TCR: T‐cell receptor
tg: transgenic
Th: T helper cell
TNF: tumor necrosis factor
WBP: whole‐body plethysmography
Penh: enhanced pause
Teff: effector T cell
Treg: regulatory T cell

## 1 INTRODUCTION

Allergic diseases represent exaggerated type 2 immune responses to otherwise innocuous, environmental antigens in genetically susceptible individuals.^1^, ^2^, ^3^, ^4^, ^5^, ^6^ The development of allergies might be caused, in part, by an imbalance between allergen‐specific T helper 2 (Th2) and T regulatory (Treg) cell responses.^3^, ^7^ Previous evidence suggests that allergic individuals may suffer from a systemic paucity of Treg cells,^8^, ^9^ which can be (partially) corrected by allergen‐specific immunotherapy that aims at alleviating allergic diseases through increasing the numbers of Tregs.^10^ In fact, Tregs have been shown to contribute to protective immune responses against allergens during normal immunity but also during allergen‐specific immunotherapy (AIT).^11^

Under physiological conditions, homeostatic Treg numbers are maintained by low level production of IL‐2 from conventional CD4^+^ T cells (Tconv).^12^ However, this balance may become disturbed upon (repeated) exposure to foreign antigens (*eg,* during infections, exposure to allergens, etc. 11, 13), resulting in exaggerated CD4^+^ Tconv cell expansion and excess IL‐2 production, which, in turn, leads to compensatory Treg cell expansion.^12^ Whether and how antigen directly contributes to Treg development and expansion has been controversially discussed in the past. While low antigen levels or impairment of TCR signaling are thought to favor peripheral Treg differentiation,^14^, ^15^, ^16^, ^17^, ^18^ high antigen levels have been reported to be required for thymic Treg development^19^, ^20^; however, also the exactly opposite was claimed recently.^21^ The matter becomes even more complex when TCR‐independent Treg expansion forces come into play, for example, those provided by strong IL‐2R and/or STAT5 agonists.^22^ In these situations, antigen seems to be entirely dispensable for sustained Treg cell expansion.^22^

Interleukin(IL)‐2 complexed to distinct anti‐IL‐2 antibodies represents such a strong IL‐2R agonist.^23^, ^24^ Distinct anti‐IL‐2 mAbs, which bind to functionally important epitopes of IL‐2, not only increase the half‐life of IL‐2 but also direct the selective expansion of certain T cell subsets, most prominently Tregs.^23^, ^25^ This would suggest IL‐2/αIL‐2 complexes as a treatment option for allergic diseases. Indeed, a recent prospective study on childhood asthma showed a correlation between low IL‐2 responses early in life and elevated total IgE levels and allergic rhinitis later on.^26^ This highlights the importance of sufficient IL‐2 levels to support peripheral tolerance induction.^12^ However, individuals suffering, for example, from perennial allergies would have to undergo IL‐2/αIL‐2 complex‐based Treg expansion in the constant presence of allergen exposure. Whether and how IL‐2/αIL‐2 complex‐based expansion of allergen‐specific Treg would be influenced by the presence of allergens remained enigmatic so far.

Therefore, we here evaluated in a humanized preclinical model of allergy the co‐administration of IL‐2/αIL‐2 complexes and allergen and how this would impact on the course of the allergic disease. We took advantage of a recently established humanized allergy model, which is specific for mugwort allergy and based on the co‐expression of a human TCR and HLA‐DR1.^4^ Mice were exposed to IL‐2/αIL‐2 complexes 23 in the presence or absence of mugwort pollen extract (MPE, days 0‐2), while control mice were exposed to MPE or PBS alone. Subsequently, we investigated whether the different treatments would lead to differences in the numbers and phenotypes of Tregs,^27^ and whether such pretreatment would impact on AHR and T‐cell effector functions in lungs upon allergen challenge. Furthermore, we wanted to elucidate the specificity of the described Treg markers CD25, Foxp3, CD73, GARP, GITR, NRP1, CTLA‐4, Helios, LAG3, CD39, PD‐1, IL‐12p35 and LAP in such expanded Treg populations and whether they are useful to define those cells.^27^

## 2 MATERIALS AND METHODS

### 2.1 Preparation of MPE

*Artemisia vulgaris* pollen (Greer, Lenoir, NC, US) was used for preparation of mugwort pollen extract (MPE) as described previously.^4^ Briefly, 10 g of mugwort pollen were incubated in 100 mL of PBS under constant stirring at 4°C for 24 hours. After centrifugation at 52 000 *g* at 4°C for 60 minutes, the supernatants were filtered and dialyzed (Spectra/Por Dialysis Membrane, MWCO: 6‐8000, Spectrum Laboratories) against PBS for 48 hours. Total protein concentration was determined by standard procedures (BCA‐bicinchoninic acid protein Kit, Pierce). The lipopolysaccharide (LPS) content of the mugwort pollen extract was ≤0.024 U/mg. Extracts were lyophilized and aliquots stored at −80°C.

### 2.2 Preparation of IL‐2/αIL‐2‐complexes

IL‐2 complexes were generated by incubating 1 µg murine recombinant IL‐2 (Peprotech) with 5 µg αIL‐2 antibody JES6‐1A12 (Life Tech Austria) at 37°C for 30 minutes.

### 2.3 Preparation of MPE immunization solution

Immunization solution was prepared by incubation of 50 µg MPE in the presence of 75 µL aluminum hydroxide (alum; 5.9‐7.1 mg/mL SERVA Electrophoresis GmbH) and 60 µL PBS w/o Ca^2+^ and Mg^2+^ at RT on a shaker for 30 minutes and subsequently used for in vivo application.

### 2.4 Mice and animal experimental procedures

TCR/DR1 transgenic mice^4^ (>12‐times backcrossed to C57BL/6J) were housed in a conventional animal facility at the Institute of Immunology (Medical University Vienna). Age‐matched female mice (6‐8 weeks of age) were used for experiments. Mice received food and water ad libitum. Sentinel mice were screened for and found free of mouse pathogenic viruses, bacteria, and parasites according to FELASA 2014 recommendations.^28^ Experimental procedures were reviewed and approved by the Institutional Review Board of the Medical University Vienna and approved by the Federal Ministry of Science, Austria (BMWF‐66.009 0118‐II 3b 2012).

### 2.5 In vivo expansion of Tregs by IL‐2/αIL‐2 complexes

TCR/DR1 transgenic mice were treated i.p. with IL‐2/αIL‐2 complexes (1 µg mIL‐2 complexed to 5 µg αIL‐2) on three consecutive days alone or in combination with MPE (50 µg/d). Treatment of mice with PBS or antigen alone was used for control groups.

### 2.6 Statistics

Data were analyzed using the GraphPad Prism 6 (La Jolla, CA, US) software. Unless indicated, all values indicate mean values ± SEM. Multiple *t* tests using the Holm‐Sidak method were used in Figure 2C and D, Figure 4C, and Figure S4A‐C. Unpaired *t* test was used for comparisons between two groups in Figure 3B. Two‐way ANOVA followed by Dunnet's correction was used for repeated measurements (Figures 5 and 6). *P* values < .05 were considered significant.

Further experimental details are provided in the Materials and Methods section in this article's Supporting information.

## 3 RESULTS

### 3.1 Phenotypic characterization of Treg generated in vivo upon exposure to IL‐2/αIL‐2 complexes in the presence or absence of antigen

**Figure 1:**
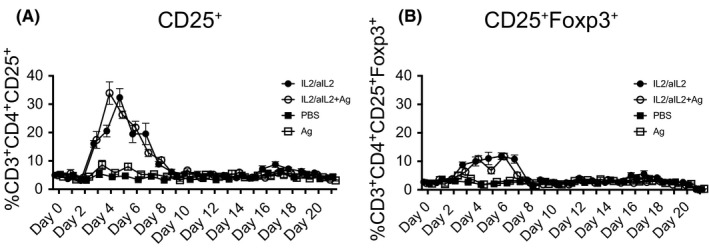
Expansion kinetics of Tregs. Shown are (A) CD3^+^CD4^+^CD25^+^ and (B) CD3^+^CD4^+^CD25^+^Foxp3^+^ PB T cells in mice treated with IL‐2/αIL‐2 complexes in the presence or absence of specific allergen. Shown are the percentages (mean ± SEM) of CD3^+^CD4^+^CD25^+^ and CD3^+^CD4^+^CD25^+^Foxp3^+^ PB T cells from days 0‐21 derived from mice i.p. injected with IL‐2/JES6‐1 complexes in the absence (IL‐2/αIL‐2) or presence of alum‐adsorbed MPE (IL‐2/αIL‐2 + MPE). Control groups received PBS (PBS) or allergen (MPE) alone. Data show the summary of two independently performed experiments with n = 6 for IL‐2/αIL‐2 and IL‐2/αIL‐2 + MPE, n = 5 for MPE and n = 4 for PBS mice per group

We here tested whether and how IL‐2/αIL‐2 complex‐based expansion of allergen‐specific Tregs would be influenced by the systemic co‐administration of allergen according to the protocol shown in Figure S1. We found that T cells expressing the high‐affinity IL‐2 receptor α‐chain (CD25), representing the first described Treg marker on CD3^+^CD4^+^ Th cells,^29^ increased from day 2 onwards. The numbers of CD3^+^CD4^+^CD25^+^ Treg peaked at 32.3 ± 3.9% on day 5 (Table S1) (6.05 ± 0.7‐fold expansion, *P* < .001 compared with baseline) (Figure 1A) and returned to baseline levels on day 10. Injection of IL‐2/αIL‐2‐complexes in combination with allergen (MPE) led to an earlier peak already on day 4 with 34.0 ± 4.9% CD3^+^CD4^+^CD25^+^ Treg cells, and a 7.0 ± 1.1‐fold expansion (*P* < .01 compared with baseline). Peripheral blood (PB) CD3^+^CD4^+^CD25^+^ T cell numbers returned to baseline levels on day 9.

Foxp3, a Treg lineage‐specific transcription factor in mice,^30^ was also significantly upregulated in PB CD3^+^CD4^+^CD25^+^ T cells after IL‐2/αIL‐2 treatment, peaking on day 5 (6.5 ± 1.2‐fold expansion, *P* < .05 compared with baseline) and returning to baseline levels on day 10 (Figure 1B). In contrast, administration of IL‐2/αIL‐2 complexes in combination with antigen (MPE) resulted in a multi‐phasic upregulation of Foxp3 in PB CD3^+^CD4^+^CD25^+^ Treg cells, with maximal peaks on day 4 and day 6 after initial treatment (10.8 ± 3.3‐fold and 9.8 ± 2.6‐fold expansion; *P* < .05 and *P* < .05 compared to baseline, respectively). Foxp3 expression returned to baseline levels on day 8. Control groups, treated with PBS or allergen alone, did not show changes in the percentages of CD3^+^CD4^+^CD25^+^ or CD3^+^CD4^+^CD25^+^Foxp3^+^ cells in PB (Figure 1).

The Treg surface markers CTLA‐4, GITR, NRP1, and LAP followed expression kinetics as CD25 upon TCR co‐engagement, ie, in the presence of allergen, with earlier but similar peak expression levels when compared to mice treated with IL‐2/αIL‐2 complexes alone (Figure S2). In contrast, peak expression levels for Helios, GARP, and CD73 were modestly lower in the presence of TCR co‐ligation (Figure S3).

**Figure 2:**
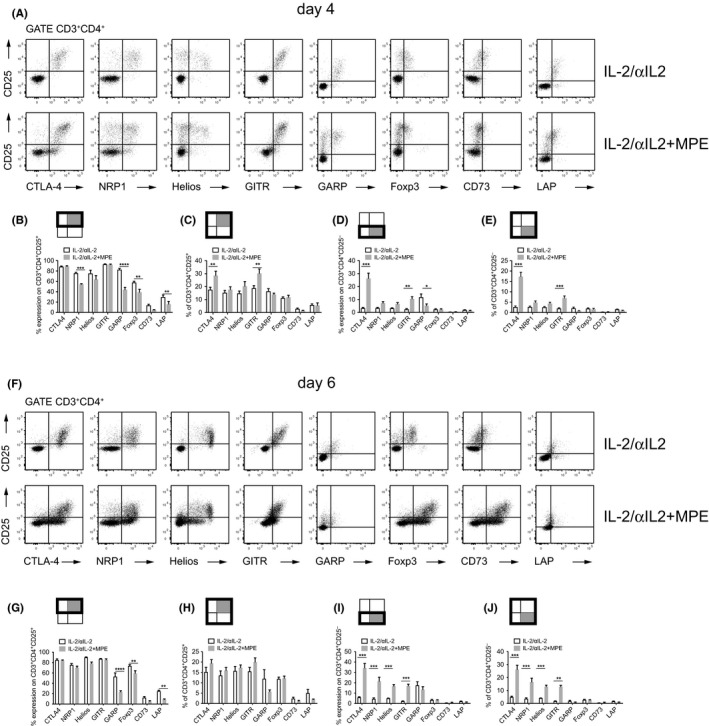
Effects of IL‐2/αIL‐2 complexes and allergen co‐exposure on Treg marker expression. Representative flow plots show CD25 versus CTLA4, NRP1, Helios, GITR, GARP, Foxp3, CD73, and LAP expression, respectively, on CD3^+^CD4^+^ PB T cells on day 4 (A) or day 6 (F) derived from mice i.p. injected with IL‐2/αIL‐2 complexes in the absence (IL‐2/αIL‐2) or presence of alum‐adsorbed MPE (IL‐2/αIL‐2 + MPE). Graphs show Treg marker positive cells (mean ± SEM) on day 4 (B‐E) and 6 (G‐J) related to either CD3^+^CD4^+^CD25^+^ T cells (B, G) or CD3^+^CD4^+^CD25^−^ T cells (D, I). Moreover, Treg marker positive and CD25^+^ (C, H) or CD25^−^ (E, J) T cells have been related to the CD3^+^CD4^+^ T cell population. The reference populations are indicated by bold frames, the tested populations by gray quadrants. Data are either representative (A and F) or show the summary (B‐E and G‐J) of two independently performed experiments with n = 6 mice per group for IL‐2/αIL‐2 and IL‐2/αIL‐2 + MPE. **P < .01, ***P < .001, ****P < .0001 comparing IL‐2/αIL‐2 and IL‐2/αIL‐2 + MPE treatment group (multiple *t* tests assuming all rows are sampled from populations with same scatter. Holm‐Sidak method was used to correct for multiple comparisons)

**Figure 3:**
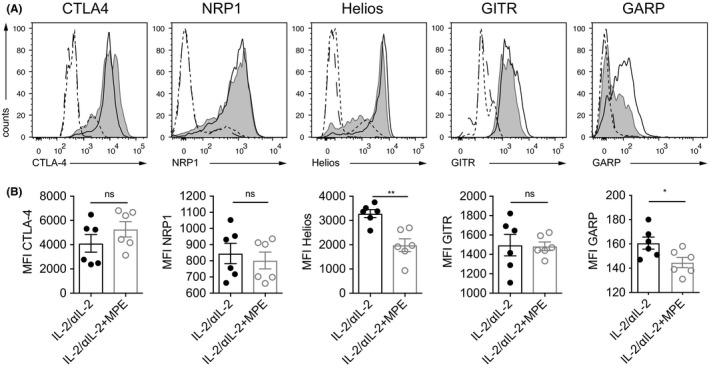
Decreased expression levels of Treg markers in the presence of allergen. A, Histogram overlays representing Treg marker expression on day 6 on CD3^+^CD4^+^CD25^+^ or PB T cells (open and gray histogram) or CD3^−^CD4^−^ lymphocytes (dotted and dashed histograms) from mice treated with IL‐2/αIL‐2 in the absence (open and dotted histogram) or presence (gray and dashed histogram) of alum‐adsorbed MPE. B, Summary plots of mean fluorescence intensity (MFI) data representative of two independently performed experiments with n = 3 mice per group for IL‐2/αIL‐2 (open bars) and IL‐2/αIL‐2 + MPE (gray bars). *P < .05, **P < .01, ***P < .001 comparing IL‐2/αIL‐2 and IL‐2/αIL‐2 + MPE treatment group (unpaired *t* test)

CTLA‐4, GITR, and GARP were found to be co‐regulated with CD25 (Figure 2A), with co‐expression on >80% of CD3^+^CD4^+^CD25^+^ T cells on day 4 (Figure 2B). In contrast, NRP1, Helios, Foxp3, and CD73 were co‐expressed only on a subset of CD3^+^CD4^+^CD25^+^ T cells (75.9 ± 4.1%; 75.4 ± 14.8%, 57.8 ± 5.9%, and 13.7 ± 5.0%, respectively (Figure 2A and B). This changed until day 6 when >90% of CD3^+^CD4^+^CD25^+^ T cells co‐expressed Helios (90.2 ± 1.9), while NRP1 and Foxp3 were still expressed on <80% of CD3^+^CD4^+^CD25^+^ T cells (Figure 2F and G). The subset of CD3^+^CD4^+^CD25^+^ T cells co‐expressing CD73 remained small (6.05 ± 1.4%) (Figure 2B and G). However, administration of IL‐2/αIL‐2 complexes in the presence of TCR ligation (MPE) significantly increased the fraction of CD3^+^CD4^+^CD25^+^ T cells, which remained negative for NRP1, GARP, and Foxp3, respectively (Figure 2A and B). In fact, NRP1, GARP, Foxp3, and LAP were found co‐expressed only on 54.6 ± 2.3%, 44.1 ± 10.6%, 39.1 ± 13.8%, and 16.1 ± 5.1 of CD3^+^CD4^+^CD25^+^ T cells, respectively, which was significantly less when compared to CD3^+^CD4^+^CD25^+^ Th cells upon exclusive treatment of mice with IL‐2/αIL‐2 complexes on day 4 (Figure 2A and B). A similar MPE‐induced reduction of marker expression on CD3^+^CD4^+^CD25^+^ T cells was observed on day 6 for GARP, Foxp3, and LAP (Figure 2F and G). In contrast to day 4, NRP1 was expressed at similar levels on day 6 when compared to mice treated in the absence of MPE allergen. Control groups (PBS or MPE) did not show such changes (Figure S4). When looking at the subset of CD25^+^Treg marker^+^ T cells as a fraction of overall CD3^+^CD4^+^ T cells on day 4, the treatment‐related differences were less apparent due to the faster expansion kinetics of Tregs in the presence of allergen (MPE), while they were evident on day 6 (Figure 2C and H). Notably, the observed effects were allergen (MPE)‐specific and not induced by alumn used as adjuvant, since no such reductions of CD25^+^Treg marker^+^ cells were observed when IL‐2/αIL‐2 complexes were administered together with BSA complexed to alumn (not shown). Moreover, TCR signaling also seemed to sustainably inhibit maximal expression levels on CD3^+^CD4^+^CD25^+^ T cells of Helios, and GARP on day 6 (Figure 3). When gating on CD3^+^CD4^+^Foxp3^+^ T cells, all Treg markers, except NRP1 (day 4) and GARP (day 6), were similarly expressed in the presence or absence of allergen (Figure S5A and B). IL‐2/αIL‐2 complexes, irrespective of the presence or absence of allergen, induced only weak expression (<5% cells) of LAG‐3, CD39, PD‐1, and IL‐12p35 on CD3^+^CD4^+^CD25^+^ Th cells (not shown).

### 3.2 Specificity of a panel of Treg marker molecules upon IL‐2/αIL‐2 complex‐based expansion of Tregs in the presence or absence of allergen

**Figure 4:**
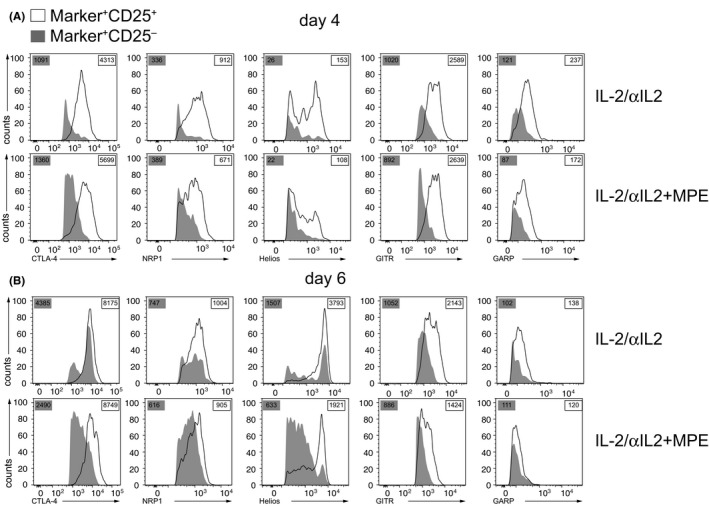
Decreased expression of Treg markers on CD3^+^CD4^+^CD25^−^ cells compared to CD3^+^CD4^+^CD25^+^ cells. Changes in Treg marker expression of (A) day 4 and (B) day 6 CD3^+^CD4^+^CD25^−^ (gray) cells and CD3^+^CD4^+^CD25^+^ (white) are shown as representative histograms for two independent experiments, each with n = 3 mice per group. Values in gray boxes indicate the geometric mean fluorescence intensity of CD3^+^CD4^+^CD25^−^ in contrast to values in white boxes for CD3^+^CD4^+^CD25^+^ cells

Next, we evaluated the Treg‐specificity of CTLA‐4, NRP1, Helios, GITR, and GARP, by gating on PB CD3^+^CD4^+^CD25^−^ or Foxp3^−^ T cells. In the presence of TCR signaling, significantly larger fractions of CD3^+^CD4^+^CD25^−^ Tconv cells co‐expressed CTLA‐4, NRP1, Helios, and GITR on day four as compared to IL‐2/αIL‐2‐treated mice (Figure 2A and D, E, Table S2); however, expression levels were generally lower when compared to CD3^+^CD4^+^CD25^+^ T cells (Figure 4). Similar findings were made upon gating on CD3^+^CD4^+^Foxp3^−^ Tconv cells in which, upon TCR ligation, a significantly larger fraction co‐expressed CTLA‐4, GITR, and NRP1, while Helios expression was unaffected on day 4 (Figures S5C and Figure S6A, Table S3). Differential expression became even more pronounced on day 6, irrespective of gating on either CD3^+^CD4^+^CD25^−^ (Figure 2F and I, J, Table S2) or CD3^+^CD4^+^Foxp3^−^ Tconv (Figure S5C, Table S3). Notably, expression levels of Treg markers on CD3^+^CD4^+^Foxp3^‐^ were generally lower when compared to CD3^+^CD4^+^Foxp3^+^ T cells (Figure S6).

These analyses suggested that neither CTLA‐4, nor GITR, Helios or NRP1 can be regarded as *bona fide* Treg markers upon IL‐2/αIL2 complex‐based Treg expansion in the presence of TCR ligation. However, in the absence of TCR ligation, ie, upon exclusive IL‐2/αIL‐2 complex‐based expansion of Tregs in vivo, these makers were almost exclusively expressed on all (or subsets) CD25^+^ and/or Foxp3^+^ CD3^+^CD4^+^ Th cells. GARP was confirmed herein as a specific Treg cell marker molecule, being co‐expressed with CD25 on CD3^+^CD4^+^ T cells when compared to CD3^+^CD4^+^CD25^−^ T cells, irrespective of whether Treg expansion was promoted by IL‐2/αIL‐2 complexes alone or in the presence of TCR ligation (88.2 ± 6.1% vs 94.7 ± 3.2% on day 4; and 82.4 ± 8.4% vs 86.6 ± 7.0% on day 6).

### 3.3 Treg cells initially expanded by IL‐2/αIL‐2 complexes recirculate more efficiently upon allergen challenge in vivo at later time points

**Figure 5:**
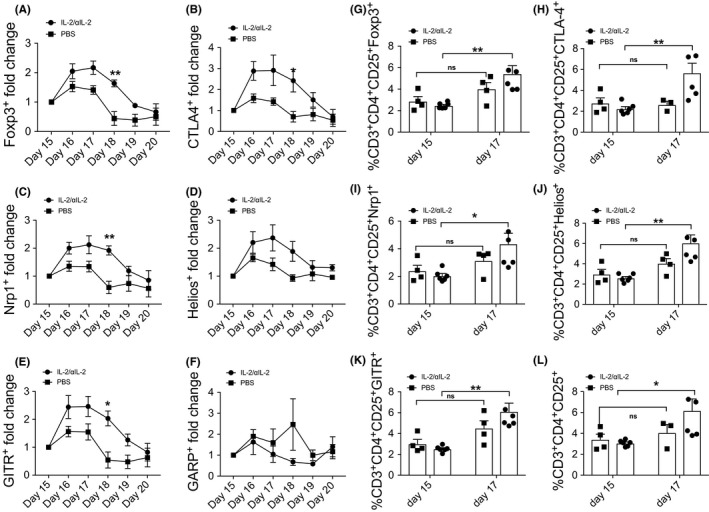
Recall Treg response in IL‐2/αIL‐2 complex‐treated mice after allergen challenge. A‐F, Fold increase and (G‐L) frequency of CD3^+^CD4^+^CD25^+^, CD3^+^CD4^+^CD25^+^Foxp3^+^, CD3^+^CD4^+^CD25^+^Helios^+^, CD3^+^CD4^+^CD25^+^GITR^+^, CD3^+^CD4^+^CD25^+^NRP1^+^, and CD3^+^CD4^+^CD25^+^CTLA4^+^ PB T cells derived from mice i.p. injected with IL‐2/αIL‐2 complexes after i.n. allergen challenge with MPE (1,5% (w/v), 450 μg per mouse). Data are pooled from two independently performed experiments with n = 6 mice for IL‐2/αIL‐2 and n = 4 mice for PBS. *P < .05, **P < .01 comparing IL‐2/αIL‐2 and PBS treatment group using two‐way ANOVA (Sidak‘s multiple comparison test)

Next, we tested whether initial expansion of Tregs with IL‐2/αIL‐2 complexes has long‐term effects. For that purpose, mice were i.n. challenged with MPE on days 13‐15 and subsequently exposed to MPE aerosol followed by daily determination of PB T cell phenotypes (Figure S1). Using day 15 as baseline, IL‐2/αIL‐2 complex‐treated mice showed a significant increase of recirculating CD3^+^CD4^+^CD25^+^ T cells co‐expressing the Treg marker molecules Foxp3, CTLA‐4, NRP1, Helios and GITR, but not GARP, respectively, already on day 16 (Figure 5A‐F), which peaked and became significant on day 17 (Figure 5G‐L and Figure S7) and continued to be clearly elevated through day 19 (Figure 5). For instance, CD3^+^CD4^+^Foxp3^+^ Treg showed a 2.2 ± 0.2‐fold expansion (increasing from 2.5 ± 0.3% on day 15 to 5.4 ± 1.8% on day 17; *P* < .01) in PB with similar increases found for other Treg marker molecules (Table S4). Interestingly, mice treated with IL‐2/αIL‐2 complexes in the presence of TCR signaling (*ie,* upon co‐administration of allergen), comparable to mice treated with PBS or allergen alone, did not show similar increases of PB Treg cell numbers after MPE challenge.

### 3.4 Exclusive treatment of mice with IL‐2/αIL‐2 complexes alleviates AHR upon challenge with allergen 2 weeks later

**Figure 6:**
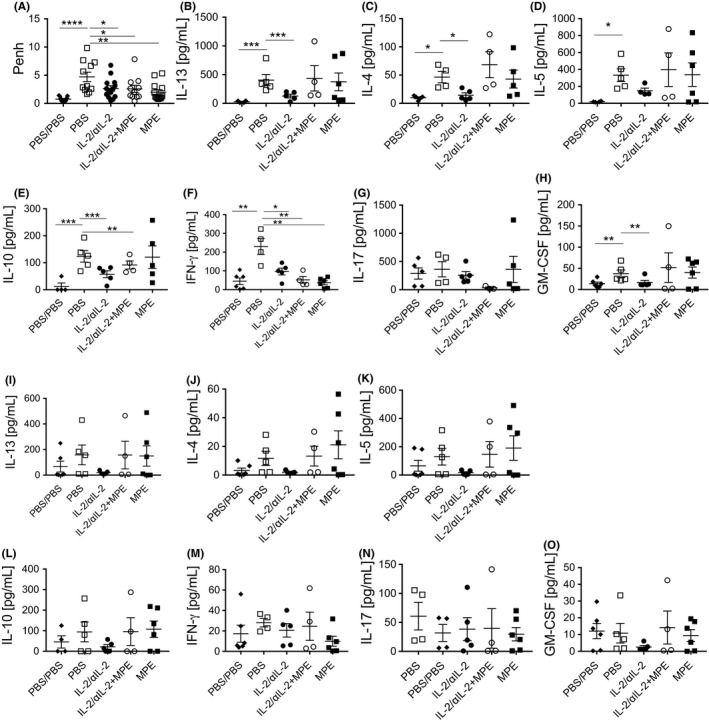
IL‐2/αIL‐2 complex treatment protects against allergic immune responses. A, Allergen‐specific airway hyperreactivity was determined after the last challenge. Using whole‐body plethysmography. Shown are mean enhanced pause (Penh) values for each mouse (individual symbols). Lung single cell suspensions were restimulated with PHA (0.15 µg/mL) (B‐H) or MPE (100 µg/mL) (I‐O) for 3 d, and supernatant levels of cytokines were determined using multiplex cytokine analyses. Data are pooled data of three independent experiments with a total of n = 15 mice for IL‐2/αIL‐2, n = 11 mice for IL‐2/αIL‐2 + MPE, n = 13 mice for PBS, n = 11 mice for MPE, and 11 mice for PBS/PBS shown in A. B‐O, Pooled data of two independent experiments combining cytokine release data from pooled mouse lung cells data. *P < .05, **P < .01, ***P < .001, ****P < .0001 One‐way ANOVA using Dunett's multiple comparison test (A) and multiple *t* test with welch correction due to the differences in variance between the study groups corrected by Bonferroni's method for multiple testing (B‐O)

Finally, we analyzed the impact of IL‐2/αIL‐2 complex treatment on lung inflammation and function upon intranasal‐followed by aerosol‐based allergen challenge (Figure S1). Notably, BAL fluid cellularity was lowest in the IL‐2/αIL‐2 pretreatment group, which was paralleled by low numbers of eosinophils, neutrophils and T cells as determined by flow cytometry (Figure S8A) and morphologically (Figure S8B). In line, mice in which Treg numbers had been increased by virtue of IL‐2/αIL‐2 complexes but not PBS‐treated control mice presented with a significantly mitigated hyperreactivity response to allergen challenge (Figure 6A). Interestingly, also those mice which had been systemically exposed to allergen, ie, in the presence or absence of co‐administered IL‐2/αIL‐2 complexes, were protected from allergen re‐exposure since they did not display increased AHR upon challenge. This finding might be explained by high‐dose tolerance induction as shown previously during rush immunotherapy.^31^ In order to correlate the observed reduction of AHR in mice treated with IL‐2/αIL‐2 complexes in the absence or presence of TCR ligation (*ie,* upon co‐administration of allergen) with possible changes in the cytokine signatures of Th cells, cytokine levels determined upon polyclonal (Figure 6B‐H) and allergen‐specific (Figure 6I‐O) re‐stimulation were analyzed in lung single cell suspensions on day 24. Notably, only the IL‐2/αIL‐2 complex‐treated mice, but not mice treated with allergen in the presence or absence of IL‐2/αIL‐2 complexes, showed reduced levels of IL‐13, IL‐4, and IL‐5 upon both polyclonal and allergen‐specific re‐stimulation of lung cells (Table S5). In contrast, the secretion of the Th1 signature cytokine IFN‐γ was found to be inhibited for all but the PBS treatment groups upon polyclonal but not allergen‐specific re‐stimulation. IL‐17 und GM‐CSF were not found to be modulated.

## 4 DISCUSSION

Treg cell‐based suppression of allergen‐specific Tconv cells is critical for the prevention and therapy of allergies.^32^, ^33^ Since allergens represent exogenous antigens, tolerance induction to them takes place in the periphery and is either Tconv intrinsic, for example, based on anergy induction which can be overcome by exogenous IL‐2,^31^, ^34^ or may be established by the expansion of populations of T regulatory cells impacting on Tconv cells.^31^ The current study demonstrates that treatment with IL‐2/αIL‐2 complexes sustainably changes the phenotype and function of peripheral Th cells, induces a wave of circulating Treg cells, which can be recalled upon allergen challenge and alleviates allergen‐induced lung pathology long after initial treatment. While it has been clearly shown that TCR signaling is not absolutely required for Treg cell division and expansion—provided that other strong Treg expansion stimuli are present, such as those delivered by IL‐2R agonists or STAT5 activators^22^—our data suggest that the presence of allergen clearly alters the phenotype of expanded Treg cells, which divides the expanded CD3^+^CD4^+^CD25^+^ cell population in those co‐expressing or lacking other *bona fide* Treg marker molecules such as NRP1, GARP, and Foxp3 (Figure 2A and B). Moreover, we demonstrate that several Treg marker molecules lack specificity and become regularly co‐expressed on Tconv when IL‐2R agonists are administered together with allergen, GARP being a notable exception.

Specifically, we found that administration of IL‐2/αIL‐2 complexes was absolutely required for substantial expansion of PB Tregs. While administration of IL‐2/αIL‐2 complexes increased the fraction of CD3^+^CD4^+^CD25^+^Foxp3^+^ T cells among PB T cells from 2.3 ± 0.6% to 16.5 ± 3.9%, the sole administration of allergen on three consecutive days was insufficient to induce significant PB Treg cell expansion as monitored by CD25 and Foxp3 co‐expression and the expression of a collection of more recently established putative Treg markers^27^ (Figure 1 and Figures S2 and S3). Although concomitant TCR ligation by allergen accelerated the appearance of peak Treg levels in PB by one day, this treatment also seemed to significantly inhibit the co‐expression of distinct Treg marker molecules on CD3^+^CD4^+^CD25^+^ and/or CD3^+^CD4^+^CD25^+^Foxp3^+^ Treg cells (Figure 2 and Figure S5). For instance, the uniform expression of NRP1 on the majority of CD3^+^CD4^+^CD25^+^ T cells upon treatment of mice with IL‐2/αIL‐2 complexes alone was clearly altered in the presence of allergen (Figure 2).

Moreover, we revealed that co‐administration of allergen (TCR ligation) induced significant expression of some Treg marker molecules on Tconv cells, defined by negativity for the canonical Treg maker molecules CD25 and Foxp3 (Figure 2 and Figure S5C). For instance, a considerable fraction (26.3 ± 10.0% to 34.3 ± 10.7%) of antigen plus IL‐2/αIL‐2 complex‐activated Tconv cells co‐expressed CTLA‐4. This was not entirely unexpected, since CTLA‐4 has initially been characterized as a T cell activation antigen^35^, ^36^ before its role as a major co‐inhibitory molecule on previously activated T cells was elucidated.^37^, ^38^ Surprisingly, however, under similar conditions, Tconv cells also co‐expressed NRP1,^39^ Helios,^40^ and GITR,^41^ all of which have been regarded as more or less strict Treg‐restricted marker molecules so far (Figure 2). Thus, our findings suggest that a number of *bona fide* Treg marker molecules rather qualify as “activation antigens” under conditions of both TCR and IL‐2R signaling. Notably, however, their expression levels on Tconv cells were generally lower when compared to the ones on CD3^+^CD4^+^CD25^+^ and/or CD3^+^CD4^+^Foxp3^+^ Treg cells (Figure 4 and Figure S6). Among the Treg markers under investigation, glycoprotein‐A repetitions predominant (GARP) was an exception since allergen exposure did not lead to its elevated co‐expression on Tconv cells. Thus, and apart from CD25, GARP was identified as the most specific surface‐expressed Treg marker analyzed in this study. Upon treatment with IL‐2/αIL‐2 complexes and allergen, not all of the analyzed CD3^+^CD4^+^CD25^+^ T cells co‐expressed GARP (Figure 2A and B). However, this was not unexpected since previous reports have shown that GARP identifies the subset of highly suppressive, activated Foxp3^+^ Treg cells.^42^, ^43^ Apart from Treg, the type I transmembrane protein GARP, which binds and activates latent TGF‐β isoforms, is typically expressed also on platelets.^42^, ^43^ Both Treg and platelets contribute to the establishment of immune tolerance^44^ with missense mutations of GARP being associated with atopic dermatitis.^45^

Another intriguing finding of our study was the fact that repeated allergen exposure via the airways was able to “recall,” at least partially, previously expanded Treg cells back into circulation (Figure 5 and Figure S7), while concomitant TCR ligation abrogated this phenomenon. Exclusive IL‐2R agonist signaling seems to initiate sustained changes in the previously expanded Treg cell compartment, which allows for their rapid (within 3 days) recall and enhanced recirculation upon exposure to allergen. Why does co‐ligation of the TCR abrogate/antagonize this recirculation/expansion capability? It is tempting to speculate that the initial presence of allergen may target Treg cells to distinct compartments within the body, which might restrict recall responses triggered by allergen challenge of the airways to compartments other than the PB. Alternatively, simultaneous TCR triggering along with IL‐2R engagement might weaken the “Treg imprint,” by favoring TCR‐ instead of IL‐2R‐dependent signaling pathways. Possible indications for such a mechanism are the significantly increased numbers of CD3^+^CD4^+^CD25^+^ T cells which lack co‐expression of the Treg marker molecules GARP, NRP1, and Foxp3. Moreover, and in light of the significant activation in the presence of antigen of Tconv, the circulatory behavior and/or expansion of Treg cells might also become influenced by the effector cytokines/receptor milieu elaborated by these cells.

Following these lines, we also observed remarkable, pretreatment‐dependent differences in the T cell target populations in the lungs after allergen challenge. In fact, in lung cells of mice which had been initially treated with IL‐2/αIL‐2 in the absence but not in the presence of allergen, Th2 cytokine levels, including IL‐13, IL‐4, and IL‐5, could only very modestly be recalled when compared to the PBS group, irrespective of whether re‐stimulation was performed polyclonally (PHA) or allergen‐specifically (MPE) (Figure 6). Similarly, sole IL‐2/αIL‐2 complex treatment reduced IL‐10 recall responses. In contrast, especially polyclonal re‐stimulation revealed a homogeneous reduction in IFN‐γ production by initially IL‐2/αIL‐2 complex but also allergen treated mice. Thus, treatment with IL‐2/αIL‐2 complexes induces long‐term tolerance against allergen challenge associated with consistent and significant reductions of Th2 cytokines produced by lung resident T cells. The present results corroborate our earlier findings indicating the disease alleviating potential of IL‐2/αIL‐2 complex‐expanded Tregs in allergy.^4^ The here‐presented data show that treatment with IL‐2/αIL‐2 complexes induces long‐term changes in the peripheral Treg cell compartment and the main target organ, that is, the lung. Our finding that application of antigen alone also leads to improvements of AHR are compatible with the induction of high‐dose tolerance as observed during rush immunotherapy previously.^31^

A possible limitation of the current study is the fact that only PB Treg populations have been monitored, while kinetic data on Treg numbers in, for example, secondary lymphoid and target organs are missing. However, the authors have opted for the precise, time‐resolved characterization of Treg expansion in PB and their marker expression profile, which would not have allowed to sample material from all organs without censoring considerably larger numbers of animals at each experimental data point.

In summary, we here show that allergen exposure alters the phenotypic composition of IL‐2/αIL‐2 complex‐expanded Treg in PB but not tolerance induction. Moreover, we identified GARP as a further (*ie,* besides CD25) reliable surface‐expressed lineage discrimination marker of IL‐2/αIL‐2 complex‐expanded Treg cells in the presence of allergen. Finally, we provide evidence that IL‐2/αIL‐2 complex‐expanded Treg cells can be recalled and suppress lung‐specific Th2 responses long after initial treatment. Further refinement of IL‐2/αIL‐2 complex‐based Treg treatment will help to influence diseases with aberrant/exuberant immune responses against foreign and endogenous antigens (ie, allergens and autoantigens).

## CONFLICT OF INTEREST

With regards to the authors disclosure of potential conflicts of interest, we would like to indicate that Winfried F. Pickl holds stocks of Biomay AG and receives honoraria from Novartis and Roche. All other authors have no additional financial interests.

## AUTHOR CONTRIBUTIONS

WFP and CK designed research; CK, US, DT, BK, and KGS performed research and analyzed data; WFP and US supervised experiments; CK, US, BK, and WFP wrote the paper.
