# Supplementary figures and images for: Allergen alters IL‐2/αIL‐2‐based Treg expansion but not tolerance induction in an allergen‐specific mouse model
Source: Allergy. 2020 Feb 15;75(7):1618–29. doi: 10.1111/all.14203 (PMC7383865; doi:10.1111/all.14203)

# Supplemental figure 1

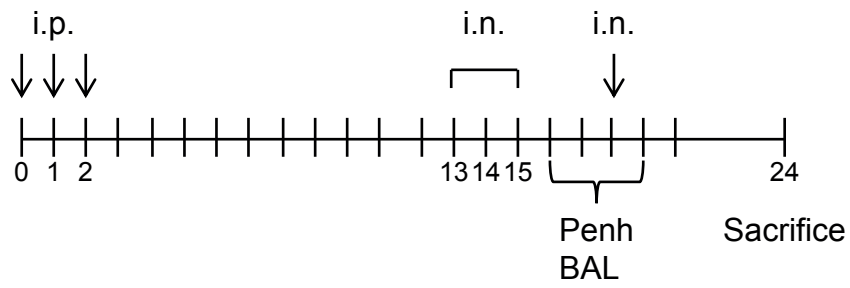

Day 0-2

1. PBS
2. PBS
3. IL-2/a-IL-2
4. IL-2/a-IL-2+MPE
5. MPE

Day 13-15

1. PBS
2. 450  $\mu$ g MPE
3. 450  $\mu$ g MPE
4. 450  $\mu$ g MPE
5. 450  $\mu$ g MPE

Supplement: Supplementary file 1 [file ALL-75-1618-s001.pdf]

Supplemental Figure 2

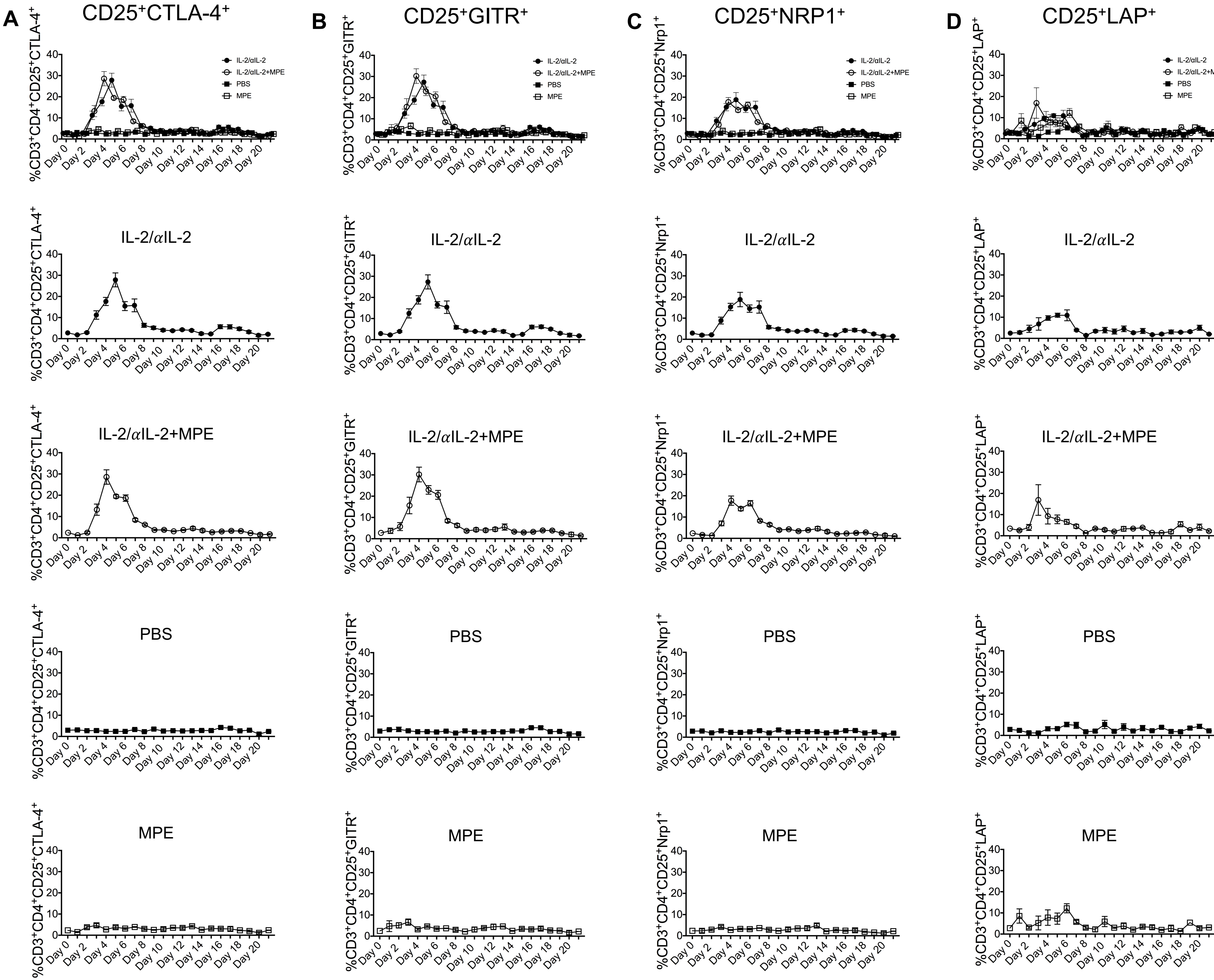

Supplement: Supplementary file 2 [file ALL-75-1618-s002.pdf]

Supplemental Figure 3

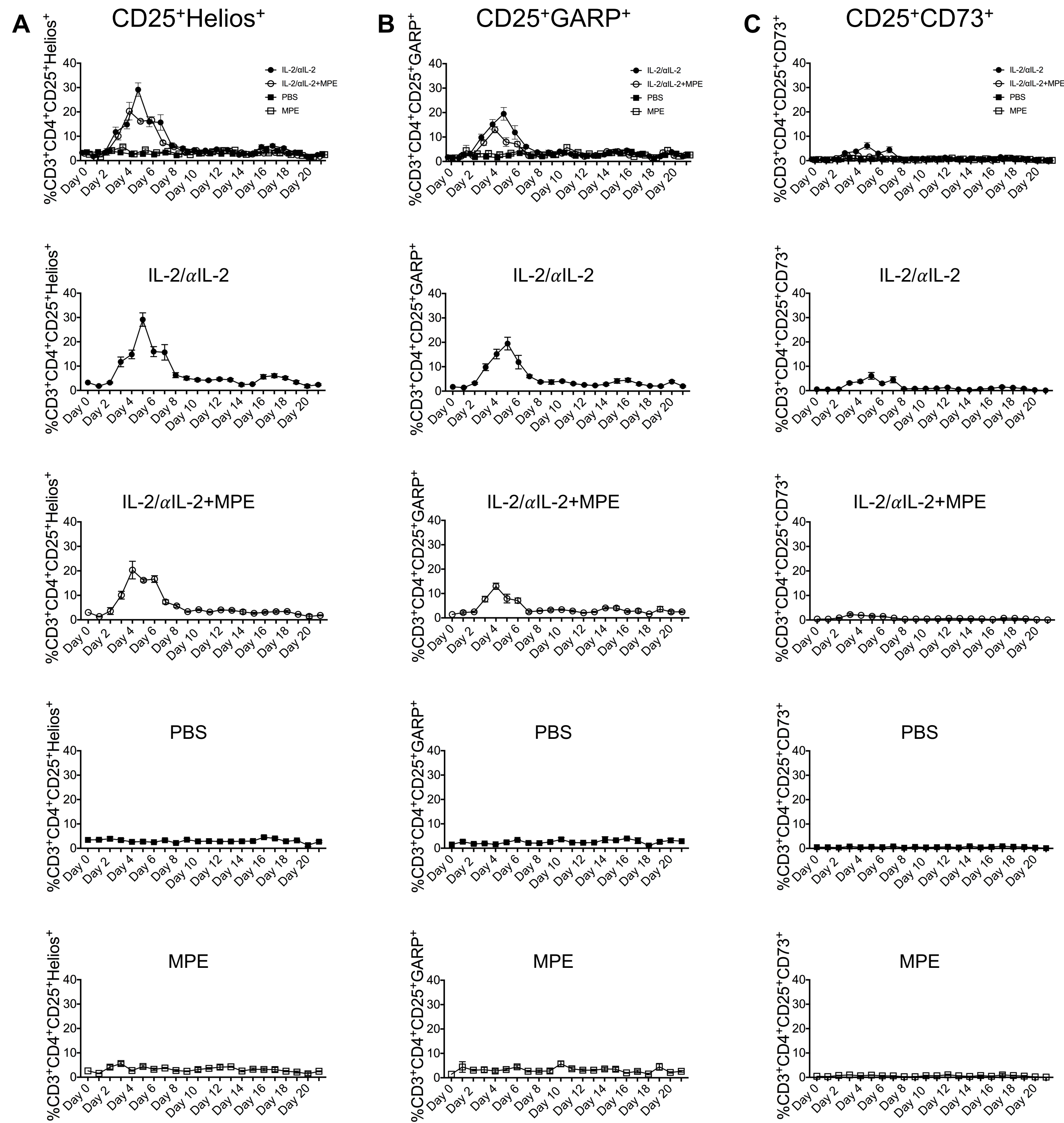

Supplement: Supplementary file 3 [file ALL-75-1618-s003.pdf]

# Supplemental figure 4

A

day 4

PBS

MPE

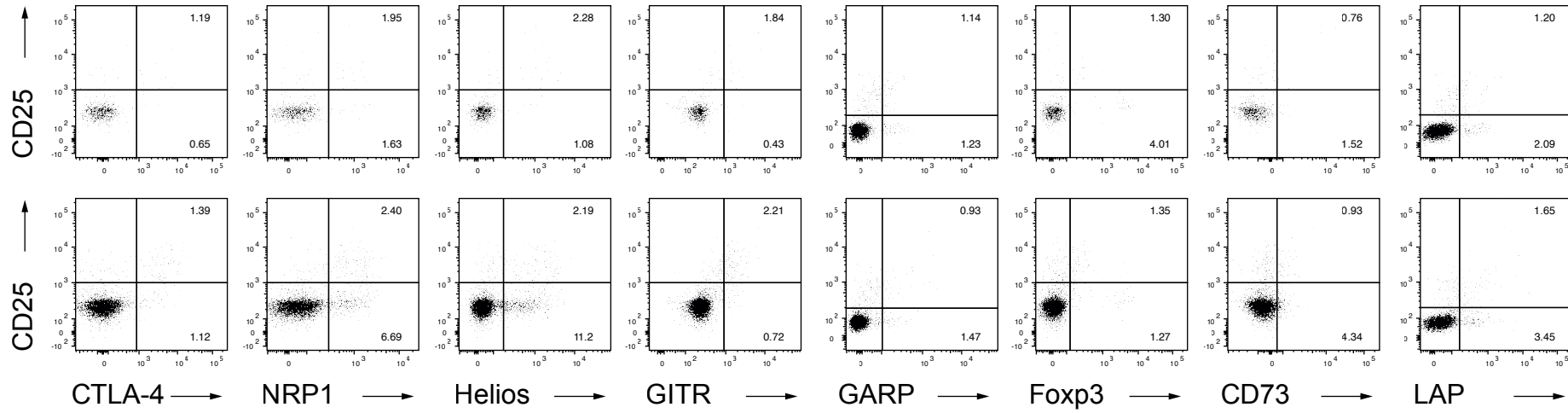

B

day 6

PBS

MPE

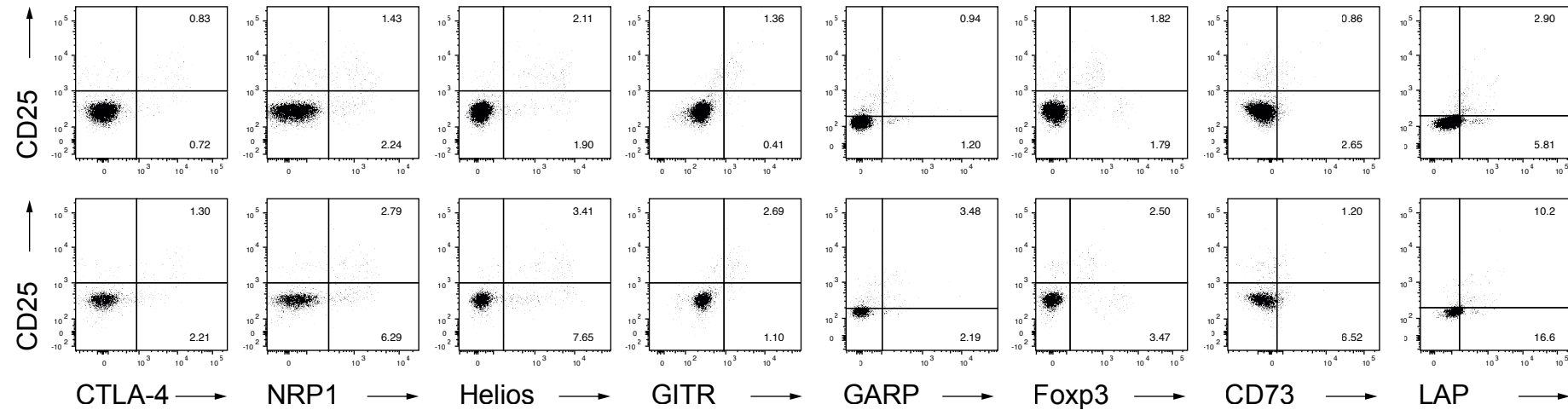

Supplement: Supplementary file 4 [file ALL-75-1618-s004.pdf]

Supplemental Figure 5

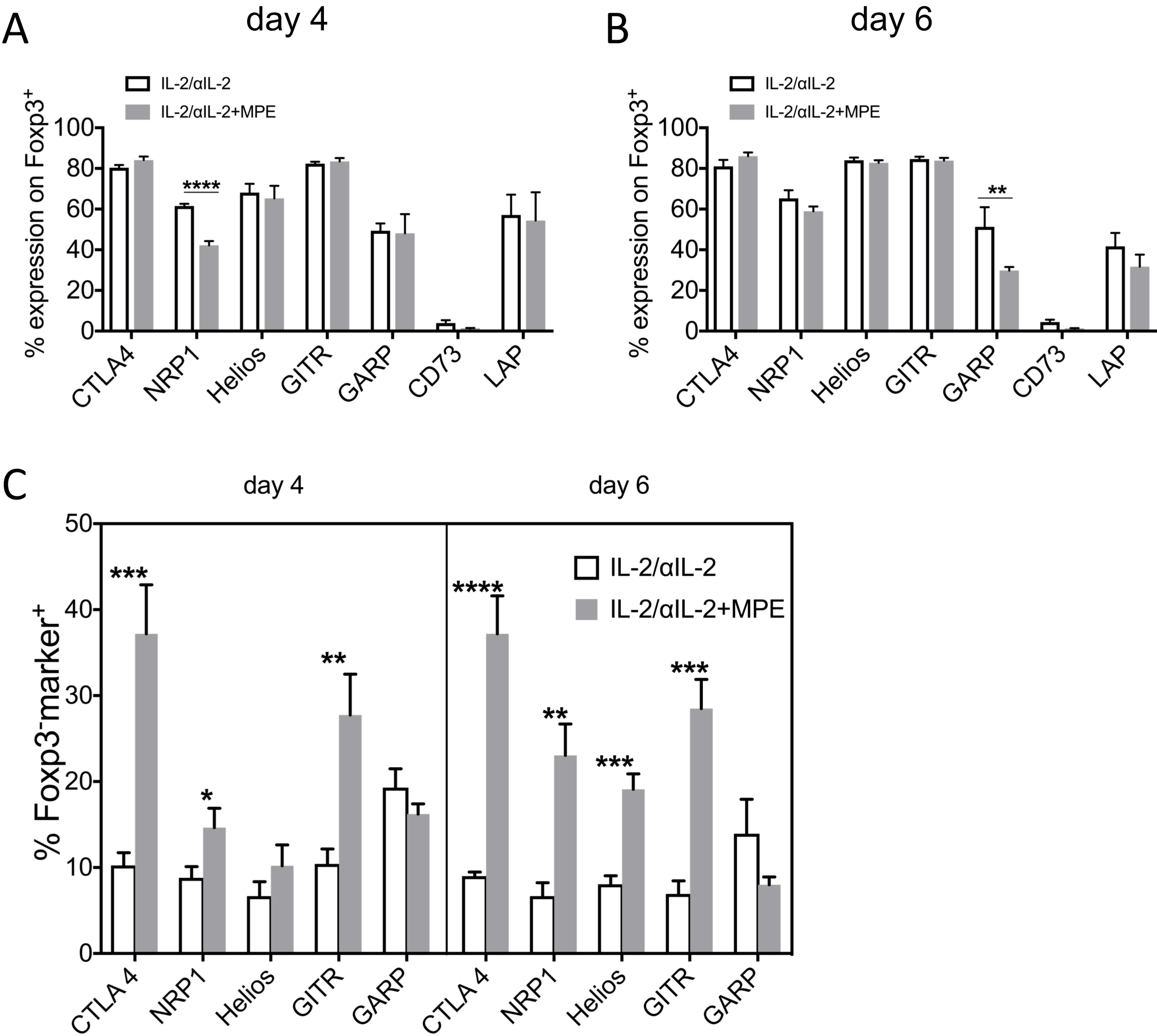

Supplement: Supplementary file 5 [file ALL-75-1618-s005.pdf]

Supplemental Figure 6

□ CD3<sup>+</sup>CD4<sup>+</sup>Foxp3<sup>-</sup>  
■ CD3<sup>+</sup>CD4<sup>+</sup>Foxp3<sup>+</sup>

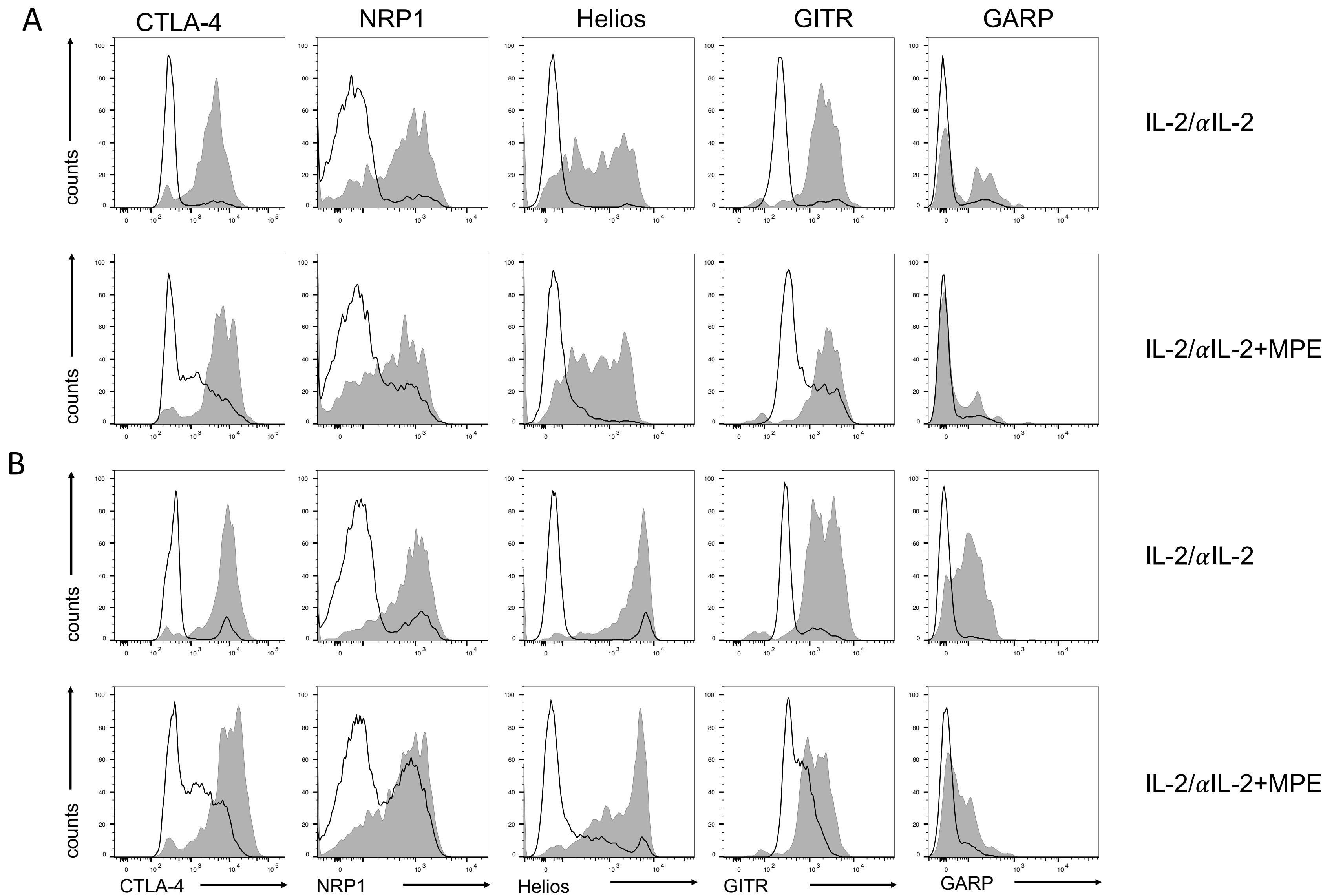

Supplement: Supplementary file 6 [file ALL-75-1618-s006.pdf]

# Supplemental figure 7

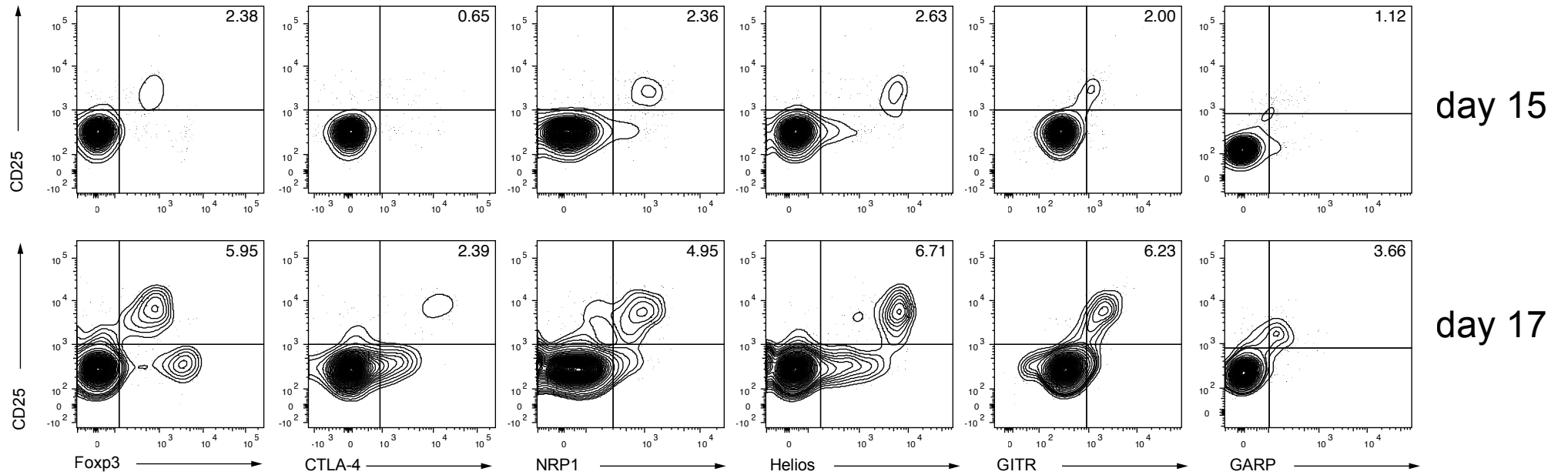

Supplement: Supplementary file 7 [file ALL-75-1618-s007.pdf]

# Supplemental figure 8

A

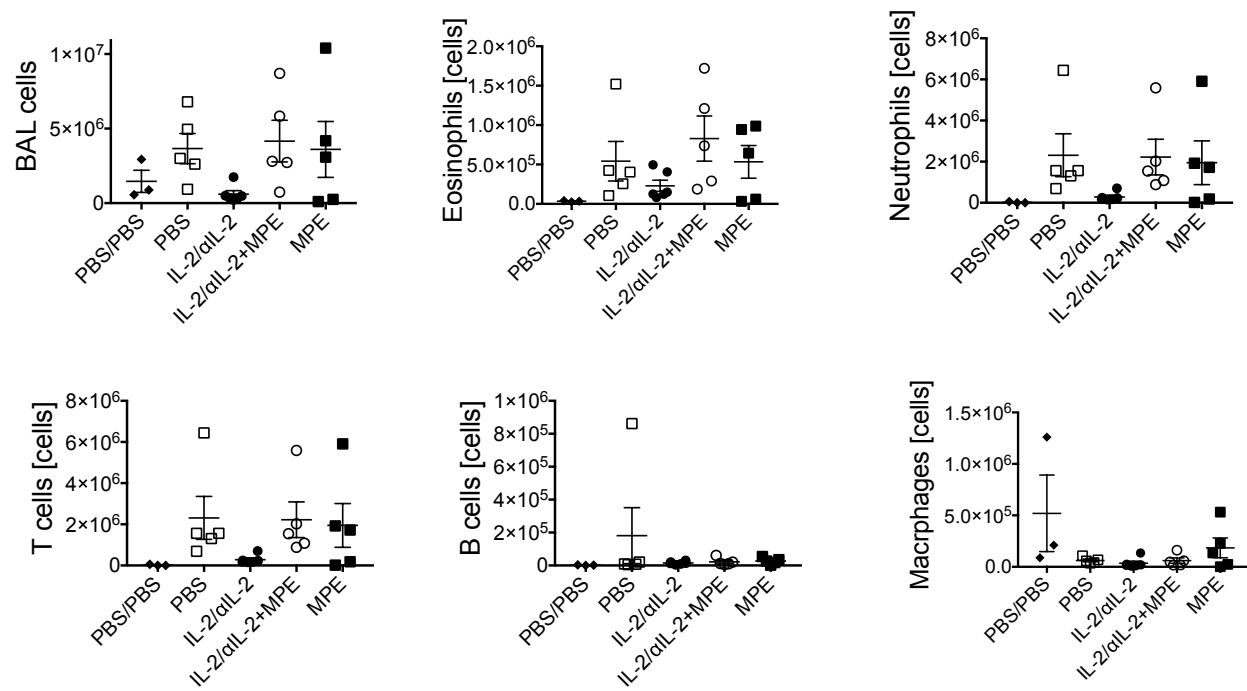

B

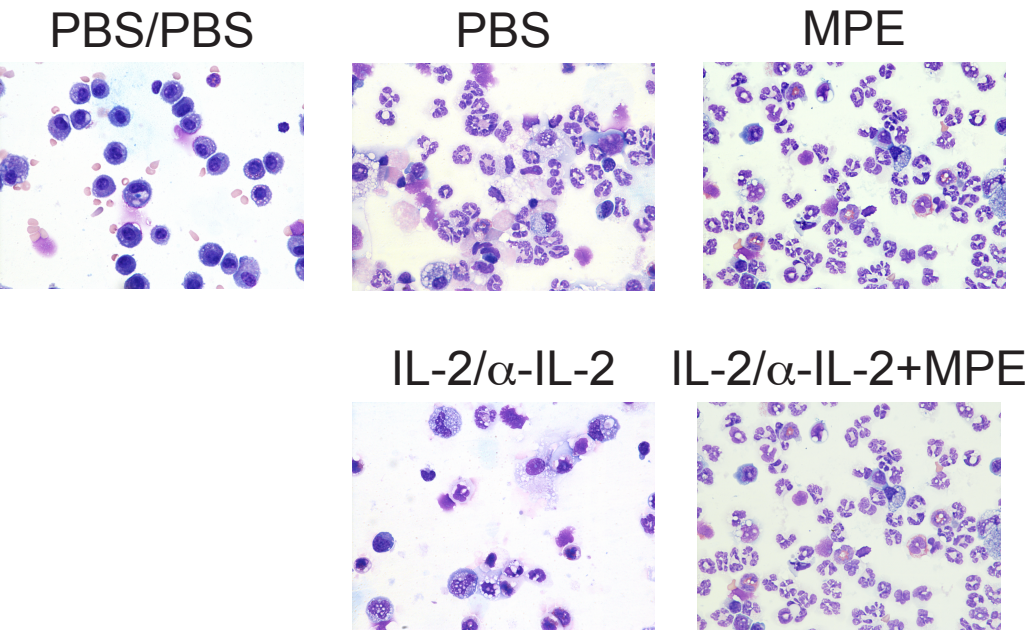

Supplement: Supplementary file 8 [file ALL-75-1618-s008.pdf]
