# Supplementary material for: Allergen alters IL‐2/αIL‐2‐based Treg expansion but not tolerance induction in an allergen‐specific mouse model
Source: Allergy. 2020 Feb 15;75(7):1618–29. doi: 10.1111/all.14203 (PMC7383865; doi:10.1111/all.14203)
Supplement: Supplementary file 9 [file ALL-75-1618-s009.docx]

**Supplemental Table 1. Comparisons of peak levels of CD25^+^ and Foxp3^+^ with baseline**

| **Gated population** | **IL-2/𝛼IL-2** |  | **IL-2/𝛼IL-2+MPE** |
| --- | --- | --- | --- |
| CD3+CD4+ CD25^+^ (%) | 5.0±1.4 vs 32.3±7.8^*^  p<0.001 |  | 5.0±1.2 vs 34.0±9.7^†^  p<0.001 |
| CD3+CD4+CD25^+^Foxp3^+^(%) | 2.7±1.3vs 11.8±2.9^§^  P<0.001 |  | 2.4±1.3 vs 11.7±2.8^§^  p<0.001 |
| CD3+CD4+CD25^+^Foxp3^+^ (MFI Foxp3) | 366±42 vs 569±173^§^  P<0.01 |  | 354±50 vs 549±262^§^  P<0.05 |
| CD3+CD4+CD25^+^ (MFI CD25) | 1974±679 vs 3485±1213^*^  p<0.05 |  | 1956±633 vs 5982±1249^†^  p<0.001 |

*) day: 0 versus 5

^†^) day: 0 versus 4

^§^) day: 0 versus 6

Data show the percentage (± SD) of CD3^+^CD4^+^ cells co-expressing CD25 or Foxp3. Alternatively, the geometric mean fluorescence intensity (MFI) after the indicated days of initial (days 0-2) IL-2/αIL-2 treatment in the presence or absence of allergen application, compared to day 0 (baseline) are shown. Data were tested with an unpaired t -test for statistical significance, respective p-values are indicated.

**Supplemental Table 2. Treg marker expression on CD3^+^CD4^+^CD25^-^ Tconv cells**

| **marker** | **day 4**  **IL-2/𝛼IL-2+MPE vs IL-2/𝛼IL-2** | | **day 6**  **IL-2/𝛼IL-2+MPE vs IL-2/𝛼IL-2** | |
| --- | --- | --- | --- | --- |
| CTLA-4 | 26.3±10% vs 3.3±1.6%^†^ | p<0.001 | 34.3±10.7% vs 6.0±1.4% | p<0.001 |
| NRP1 | 7.3±2.7% vs 3.4±1.4% | p=0.01 | 21.5±9.4% vs 4.5±2.2% | p=0.001 |
| Helios | 6.6±2.6% vs 3.2±0.9% | p=0.01 | 16.8±3.7% vs 5.0±0.6% | p<0.001 |
| GITR | 10.5±4.0% vs 2.5±1.1% | p<0.001 | 11.7±6.1% vs 2.2±0.7% | p=0.004 |
| GARP | 5.2±3.2%vs 11.8±6.1% | p=0.04 | 13.4±7.0% vs 17.6±8.4% | p=0.4 |

^†^ Data show percent ± SD of CD3^+^CD4^+^CD25^-^ Tconv cells expressing the respective Treg marker molecule after the indicated days of initial (days 0-2) IL-2/αIL-2 treatment in the presence or absence of allergen application. Data were tested with an unpaired t -test test for statistical significance, respective p-values are indicated.

**Supplemental Table 3. Treg marker expression on CD3^+^CD4^+^Foxp3^-^ Tconv cells**

| **marker** | **day 4**  **IL-2/𝛼IL-2+MPE vs IL-2/𝛼IL-2** | | **day 6**  **IL-2/𝛼IL-2+MPE vs IL-2/𝛼IL-2** | | |  |
| --- | --- | --- | --- | --- | --- | --- |
| CTLA-4 | 32.7±13.9% vs 10.2±3.6%^†^ | p=0.001 | | 37.2±10.8% vs 9.0±1.2% | p<0.001 | |
| NRP1 | 14.7±5.5% vs 8.8±3.2% | p=0.048 | | 23.0±9.0% vs 6.7±3.8% | p=0.002 | |
| Helios | 10.2±5.9% vs 6.7±4.1% | p=0.3 | | 19.1±4.4% vs 8.0±2.5% | p<0.001 | |
| GITR | 27.8±11.7% vs 10.4±4.2% | p=0.007 | | 28.5±8.3% vs 7.0±3.7% | p<0.001 | |
| GARP | 16.2±3.0% vs 19.3±5.3 | p=0.24 | | 8.0±2.2% vs 13.9±9.8% | p=0.18 | |

^†^ Data show percent ± SD of CD3^+^CD4^+^Foxp3^-^ Tconv cells expressing the respective Treg marker molecule after the indicated days of initial (days 0-2) IL-2/αIL-2 treatment in the presence or absence of allergen application. Data were tested with an unpaired t-test for statistical significance, respective p-values are indicated.

**Supplemental Table 4. Recall response after allergen challenge**

| **Marker** | **day 15** | **day 17** | **fold change** |
| --- | --- | --- | --- |
| CD25 | 3.1±0.3^†^  *3.4±1.0* | 6.2±2.7  *4.1±1.3* | 2.0  *1.2* |
| Foxp3 | 2.5±0.3  *2.9±0.9* | 5.4±1.8  *4.0±1.2* | 2.2  *1.4* |
| CTLA-4 | 2.3±0.5  *2.8±1.0* | 5.7±2.3  *2.6±0.6* | 2.5  *0.9* |
| NRP1 | 2.0±0.4  *2.4±0.8* | 4.4±1.9  *3.1±0.9* | 2.0  *1.3* |
| Helios | 2.6±0.4  *3.0±0.9* | 6.1±2.0  *4.0±0.9* | 2.3  *1.4* |
| GITR | 2.5±0.3  *3.0±0.8* | 6.1±2.0  *4.5±1.4* | 2.5  *1.5* |
| GARP | 4.1±2.3  *2.3±1.2* | 3.0±1.2  *3.5±2.6* | 0.7  *1.6* |

^†^ Data show percent ± SD of CD3^+^CD4^+^ PB T cells expressing the respective Treg marker molecule after in vivo re-challenge, data of the PBS controls are shown in italics

**Supplemental Table 5. Cytokine responses in PHA-restimulated lung single cell suspensions**

| **Cytokine** | **PBS** | **IL-2/𝛼IL-2** | **p-value** |
| --- | --- | --- | --- |
| IL-2 | 45.2±15.4^†^ | 31.65±13.4 | 0.0204 |
| IL-4 | 46.4±19.9 | 14.34±9.4 | 0.0452 |
| IL-5 | 332±167.4 | 148.1±62.3 | 0.096 |
| IL-10 | 123.8±21.3 | 57.39±12.9 | <0.001 |
| IL-13 | 404.4±219.6 | 127.2±73.2 | <0.001 |
| IFN𝛾 | 230.1±85.2 | 96.5±41.2 | 0.0312 |
| IL-17 | 361.7±135.4 | 255.6±66-7 | 0.9056 |
| GM-CSF | 37.4±8.9 | 16.6±5.1 | 0.0384 |
|  |  |  |  |

^†^ Data show cytokine levels in pg/ml ± SD. Lung cell suspensions of mice treated on days 0-2 with PBS (PBS) or IL-2/αIL-2 (IL-2/αIL-2) and i.n. challenged with MPE on days 13-15 followed by determination of AHR were restimulated on day 24 with PHA and cytokines determined after 72 hours. Data were tested by multiple T test with welch correction due to the differences in variance between the study groups and p-values were corrected according to Bonferoni’s method for the number of comparisons.

**Supplemental Table 6. List of monoclonal antibodies used for flow cytometry for analyses of cell surface antigens and transcription factors of peripheral blood leukocytes.**

| **specificity** | | **clone name** | | **species** | | **fluorophore** | | **source** |
| --- | --- | --- | --- | --- | --- | --- | --- | --- |
| CD25 | | PC61 | | rat | | FITC | | BioLegend, San Diego, CA, USA |
| CD3 | | 17A2 | | rat | | BV785 | | BioLegend, San Diego, CA, USA |
| CD39 | | 24DMS1 | | rat | | Alexa Fluor 700 | | eBioscience, San Diego, CA, USA |
| CD4 | | GK1.5 | | rat | | PerCP-eFluor 710 | | eBioscience, San Diego, CA, USA |
| CD4 | | GK1.5 | | rat | | BV510 | | eBioscience, San Diego, CA, USA |
| CD4 | | 17A2 | | rat | | PE-Cy7 | | BioLegend, San Diego, CA, USA |
| CD49b | | DX5 | | rat | | BV510 | | BioLegend, San Diego, CA, USA |
| CD73 | | TY/11.8 | | rat | | BV421 | | eBioscience, San Diego, CA, USA |
| CTLA-4 | | UC10-4B9 | | hamster | | PE Texas Red | | eBioscience, San Diego, CA, USA |
| Foxp3 | | FJK-16S | | rat | | APC | | eBioscience, San Diego, CA, USA |
| Foxp3 | | FJK-16S | | rat | | PE | | eBioscience, San Diego, CA, USA |
| GARP | | YGIC86 | | rat | | PE-Cy7 | | eBioscience, San Diego, CA, USA |
| GITR | | DTA-1 | | rat | | FITC | | eBioscience, San Diego, CA, USA |
| Helios | | 22F6 | | hamster | | PE | | eBioscience, San Diego, CA, USA |
| IL12p35 | | 4D10p35 | | rat | | APC | | eBioscience, San Diego, CA, USA |
| LAG3 | | C9B7W | | rat | | Apc-Cy7 | | eBioscience, San Diego, CA, USA |
| LAP | | TW7-16B4 | | mouse | | PerCP-Cy5.5 | | eBioscience, San Diego, CA, USA |
| NRP1 | | 3DS304M | | rat | | PE Cy7 | | eBioscience, San Diego, CA, USA |
| PD-1 | | 29F.1A12 | | rat | | BV605 | | BioLegend, San Diego, CA, USA |
| TRBV18 | | BA62.6 | | mouse | | PE | | BC, Brea, CA, USA |
| CD3 | | 17A | | rat | | FITC | BioLegend, San Diego, CA, USA | |
| CD19 | | 1D3 | | rat | | PE | BD Pharmingen, Palo Alto, CA | |
| CD11b | | M1/70 | | rat | | APC-Cy7 | BioLegend, San Diego, CA, USA | |
| CD11c | | N418 | | hamster | | PE-Cy7 | BioLegend, San Diego, CA, USA | |
| Siglec F | | S17007L | | rat | | APC | BioLegend, San Diego, CA, USA | |
| MHC II | | M5/114.15.2 | | rat | | BV650 | BioLegend, San Diego, CA, USA | |
| Ly6G | | 1A8 | | rat | | BV605 | BioLegend, San Diego, CA, USA | |

Table shows the specificity, clone names, species and supplier (source) of the respective monoclonal antibodies and their conjugation with the respective fluorophores used in this study.

**Supplemental Table 7. List of primary and secondary antibodies used for multiplex-based cytokine determinations**

| **specificity** | **clone name** | **species** | **source** |
| --- | --- | --- | --- |
| IL-2 | JES6-5H4  JES6-1A12 | rat | eBioscience, SantaClara, CA, USA |
| IL-4 | BVD6-24G2  11B11 | rat | eBioscience, SantaClara, CA, USA |
| IL-5 | TRFK4  TRFK5 | rat | eBioscience, SantaClara, CA, USA |
| IL-10 | JES5-2A5  JES5-16E3 | rat | eBioscience, SantaClara, CA, USA |
| IL-13 | eBio13A  eBio13A | rat | eBioscience, SantaClara, CA, USA |
| IL-17 | eBio17B7 eBio17CK15A5 | rat | eBioscience, SantaClara, CA, USA |
| IFN-γ | R4-6A2  AN-18 | rat | eBioscience, SantaClara, CA, USA |
| TNF-α | XT3/XT22  1F3F3D4 | rat | eBioscience, SantaClara, CA, USA |
| GM-CSF | MP1-31G6  MP1-22E9 | rat | eBioscience, SantaClara, CA, USA |

Table shows the specificity, clone names, species and supplier (source) of the respective anti-cytokine antibodies for determination of cytokine levels in cell culture supernatants used in this study.
