# Supplementary material for: Allergen alters IL‐2/αIL‐2‐based Treg expansion but not tolerance induction in an allergen‐specific mouse model
Source: Allergy. 2020 Feb 15;75(7):1618–29. doi: 10.1111/all.14203 (PMC7383865; doi:10.1111/all.14203)
Supplement: Supplementary file 10 [file ALL-75-1618-s010.docx]

**SUPPLEMENTAL INFORMATION FOR ONLINE SUPPLEMENT**

**Allergen alters IL-2/αIL-2-based Treg expansion but not tolerance induction in an allergen-specific mouse model**

#### Running title: Treg expansion by IL-2/αIL-2 complexes.

Cordula Köhler BSc^1*^, Ursula Smole PhD^1*^, Bernhard Kratzer PhD^1*^, Doris Trapin MSc^1^, Klaus G. Schmetterer^2^ and Winfried F. Pickl MD^1^

^1^Institute of Immunology, Center for Pathophysiology, Infectiology and Immunology ^2^Clinical Department of Medical and Chemical Laboratory Diagnostics, Medical University of Vienna, Vienna, Austria

* contributed equally to the work

**Corresponding author:**

Winfried F. Pickl, MD

Institute of Immunology, Center for Pathophysiology, Infectiology and Immunology, Medical University of Vienna, Lazarettgasse 19, 1090 Vienna, Austria.

Phone: (+431) 40160 33245.

Fax: (+431) 40160 933245.

Email: winfried.pickl@meduniwien.ac.at.

ORCID ID: orcid.org/0000-0003-0430-4952

**SUPPLEMENTAL MATERIALS AND METHODS**

**Tail vein sampling for whole blood**

Heparinized venous whole blood sampled by tail vein incision, according to standardized procedures. To be able to measure single cells by flow cytometry, coagulation of the whole blood samples was performed by adding 10 μl of a heparin-solution (Natrium-Heparin, 5000I.E./mL, Gilvasan®) to the collection tubes. During blood sampling animals were fixed and the tail vein was carefully cut with a sterile razor blade. After collecting 3-4 drops of venous blood, the bleeding was stopped by compression of the incision site with a sterile swab.

**Phenotypic characterization of PB Treg cells expanded in the presence of IL-2/αIL-2-complexes *in vivo***

Heparinized blood collected from individual mice and lung cell suspension were incubated for with 1 ml of a 1:10 diluted BD Pharm Lyse solution for 3 minutes, then the reaction was stopped with FACS buffer and cells were washed once by centrifugation at 500 g for 5 minutes. Cell surface antigens were stained according to standard procedures ([46](#_ENREF_46)). Briefly, cell suspensions were incubated with 30 µl of the indicated antibodies (**Supplemental Table 6)** at RT in the dark for 30 minutes. Subsequently, cells were washed once with FACS buffer. Afterwards, cell suspensions were incubated on ice, in the dark with FIX solution (1:4) (eBioscience) for 30 minutes. Next, cell suspensions were washed once with Perm solution (1:10), incubated with the desired dilutions of antibodies recognizing intracellular antigens (diluted in Perm solution) on ice in the dark for 30 minutes. After an additional wash with Perm solution followed by one wash with FACS buffer stained cell suspensions were immediately subjected to flow cytometric acquisition. Data were recorded exclusively from viable cells by using appropriate forward and side scatter gating. Analysis was performed on a Fortessa LSRII flow cytometer (Becton-Dickinson, Palo Alto, CA) followed by data analysis using the FlowJo v 9.3.3 software (Tree Star Inc, Ashland, OR).

**Allergen challenge**

TCR/DR1 mice, pre-treated with IL-2/αIL-2 complexes alone or in combination with mugwort pollen extract (MPE) and control mice pre-treated MPE or PBS were challenged i.n. either with 450 µg (1,5% (w/v) in PBS) or PBS (PBS/PBS group) on days 13, 14 and 15 after initial treatment. To facilitate administration of the extract, the mice were shortly anesthetized with isofluran (Sevofluran, AbbVie®).

**Whole body plethysmography (WBP)**

Lung function in the five groups of mice was analyzed upon challenge with 1% (w/v) mugwort-pollen extract solution by unrestrained whole body plethysmography (Buxco, Winchester, UK). Challenge consisted of an acclimation phase, a nebulization phase and a responding phase.

**Analysis of bronchoalveolar lavage fluid (BALF) cells**

BALF was collected on day 17 by flushing the airways with 1 ml of ice-cold PBS (without Ca2+ and Mg2+). Cells were collected by centrifugation at 500g for 5 min. Total leukocyte cell numbers were determined and cytospin preparations (Cytospin 4 centrifuge, Thermofisher) of BALF cells were stained using a modified Wright-Giemsa stain (HematTek Stain Pack, Siemens, Erlangen, Germany) on a Hematek slide stainer (Siemens). In parallel, BALF cells were stained with the mAb panel as described (**Supplemental Table 6**). To prevent non-specific binding to Fc receptors the 2.4G2 blocking reagent (6 μg/ml, Becton Dickinson) was added to the mAb mix. The detailed cellular composition of BALF was determined on a Becton Dickinson Fortessa flow cytometer using FACS DIVA (Becton Dickinson) and FlowJo softwares (Treestar, Costa, Mesa, CA).

**Isolation of lungs and preparation of single cell solutions**

Lung homogenates were prepared according to standard procedures. Censored mice were disinfected by soaking them in 70% ethanol for one minute. Mouse lungs were obtained after placing them backsides onto a clean surface. A midline incision was performed to expose the lungs and the heart. For that purpose, the rib cage was cut and opened widely. Lungs, heart and thymus were pulled out of the thorax, the lungs were isolated accordingly. Subsequently, lungs were chopped into 5 mm pieces and incubated with digestion solution containing 1.8 μg/ml collagenase and 40 μg/ml DNase (Sigma Chemicals) at 37°C for 60 minutes. Then, cells were disaggregated and filtered through a 70 μm nylon cell strainer (Becton Dickinson). The filtrate was centrifuged at 500 x g at 4°C for 5 minutes. The cell pellet was re-suspended in ammonium-chloride lysis buffer, incubated at room temperature for 5 minutes, washed once in 1 x IMDM before subjection to functional analyses.

**Cytokine measurements**

Lung cell suspensions were restimulated with 0.15 µg/ml phytohaemagglutinin (PHA) or MPE (100 µg/ml) and cell culture supernatants were harvested 72 hours later for cytokine analyses using the Luminex system (Luminex 100IS, Biomedica, Vienna, Austria). **Supplemental** **Table 7** show the antibodies used to determine IL-2, IL-4, IL-10, IL-13, IL-17, IFN-γ, TNF- α and GM-CSF levels in cellular supernatants.

**REFERENCES**

1. Cossarizza A, Chang HD, Radbruch A, Akdis M, Andra I, Annunziato F, et al. Guidelines for the use of flow cytometry and cell sorting in immunological studies. Eur. J. Immunol. 2017;47(10):1584-1797.

**FIGURE LEGENDS FOR ONLINE SUPPLEMENT**

**Figure S1.** Mice were injected i.p on three consecutive days (days 0, 1 and 2) with IL-2 (1 µg) complexed to JES6-1 (5 µg) or IL-2/JES6-1 plus alum-adsorbed mugwort pollen allergen (MPE, 50 µg) and analyzed for the expression of Treg markers on peripheral blood CD3^+^CD4^+^ T cells from day 0-21. Control groups received PBS or alum-adsorbed allergen alone. Two weeks after the initial treatment, mice received three challenges with MPE extract intranasally (i.n.) on days 13-15 and subsequently airway reactivity (Penh) upon exposure to aerosolized MPE was assessed. On the day of the sacrifice (day 24), organs were isolated and immune cells stained for marker expression. Lung cells and splenocytes were restimulated in the presence of MPE and cellular proliferation and cytokine production were determined.

**Figure S2.** Co-expression of (**A**) CTLA-4, (**B**) GITR, (**C**) NRP1, (**D**) LAP1 on CD3^+^CD4^+^CD25^+^ PB T cells. Shown are the percentages (mean±SEM) of CD3^+^CD4^+^CD25^+^marker^+^ PB T cells from days 0-21 derived from mice i.p. injected with IL-2/𝛼IL-2 complexes in the absence (IL-2/αIL-2) or presence of alum-adsorbed MPE (IL-2/𝛼IL-2+MPE). Control groups received PBS (PBS) or allergen (MPE) alone. Data are pooled from two independently performed experiments with n=6 for IL-2/𝛼IL-2 and IL-2/𝛼IL-2+MPE, n=5 for MPE and n=4 for PBS mice per group.

**Figure S3.** Co-expression of (**A**) Helios, (**B**) GARP, (**C**) CD73 on CD3^+^CD4^+^CD25^+^ PB T cells. Shown are the percentages (mean±SEM) of CD3^+^CD4^+^CD25^+^marker^+^ PB T cells from days 0-21 derived from mice i.p. injected with IL-2/𝛼IL-2 complexes in the absence (IL-2/𝛼IL-2) or presence of alum-adsorbed MPE (IL-2/𝛼IL-2+MPE). Control groups received PBS (PBS) or allergen (MPE) alone. Data are pooled from two independently performed experiments with n=6 for IL-2/𝛼IL-2 and IL-2/𝛼IL-2+MPE, n=5 for MPE and n=4 for PBS mice per group.

**Figure S4.** Representative flow plots show CD25 versus CTLA4, NRP1, Helios, GITR, GARP, Foxp3, CD73 and LAP expression, respectively, on CD3^+^CD4^+^ PB T cells on day 4 (**A**) or day 6 (**B**) derived from mice i.p. injected with PBS or alum-adsorbed MPE. Data are representative of two independently performed experiments with n=6 mice per group.

**Figure S5.** Effects of IL-2/𝛼IL-2 complexes and allergen co-exposure on Treg marker expression on CD3^+^CD4^+^Foxp3^-^ and Foxp3^+^ T cells. Changes in Treg marker expression of (**A**) day 4 and (**B**) day 6 CD3^+^CD4^+^Foxp3^+^ cells. (**C**) Treg marker expression on CD3^+^CD4^+^Foxp3^-^ T cells on day 4 and day 6. Shown are the percentages (mean±SEM) of CD3^+^CD4^+^Foxp3^-^ cells expressing the respective markers as indicated. Data are pooled from two independently performed experiments with n=6 for IL-2/𝛼IL-2 and IL-2/𝛼IL-2+MPE mice per group. ****P*<0.01, ****P*<0.001, ****P <0.0001 comparing IL-2/𝛼IL-2 and IL-2/𝛼IL-2+MPE treatment group (multiple t-tests assuming all rows are sampled from populations with same scatter. Holm-Sidak method was used to correct for multiple comparisons).

**Figure S6.** Effects of IL-2/𝛼IL-2 complexes and allergen co-exposure on Treg marker expression on CD3^+^CD4^+^Foxp3^-^ and CD3^+^CD4^+^Foxp3^+^ T cells. Histogram overlays representing Treg marker expression on (**A**) day 4 and (**B**) day 6 derived from mice i.p. injected with IL-2/𝛼IL-2 complexes in the absence (IL-2/𝛼IL-2) or presence of alum-adsorbed MPE (IL-2/𝛼IL-2+MPE). CD3^+^CD4^+^Foxp3^-^ cells (open histograms); CD3^+^CD4^+^Foxp3^+^ T cells (grey histograms). Flow plots are representative of n=6 mice per group.

**Figure S7**. Recall Treg response in IL-2/αIL-2 complex-treated mice after allergen challenge**.** Shown are representative contour plots of PB (day 15 and day 17) of mice pre-treated with IL-2/𝛼IL-2 complexes (i.p., days 0-2) and subsequently i.n. allergen-challenged with MPE (days 13-15). Numbers in the upper left quadrant indicate the fraction of CD3^+^CD4^+^CD25^+^marker^+^ T cells. Flow plots are representative of n=6 mice.

**Figure S8**. BAL cell analyses of differentially pre-treated mice**. (A)** Shown are absolute BAL cell numbers and numbers of eosinophils, neutrophils, T cells, B cells and macrophages of mice treated as indicated determined by flow cytometry. (**B)** Shown are representative cytospin preparations of the respective treatment groups. Shown is the summary (A) or representative cytospins (B) of 6 mice (except 5 for IL-2/aIL-2 plus MPE, MPE, PBS and 3 for PBS/PBS).
